# The Oncogenic Burden of Obesity: Mechanistic Links Between Adiposity and Gastrointestinal Cancers—A Comprehensive Narrative Review

**DOI:** 10.3390/biomedicines13071571

**Published:** 2025-06-26

**Authors:** Felicia Lee, Jessica Moore, Mariam Markouli, Wissam Ghusn

**Affiliations:** 1 Department of Internal Medicine, Boston University, 801 Massachusetts Ave, Boston, MA 02119, USA; felicia.lee@bmc.org (F.L.); jessica.moore@bmc.org (J.M.);; 2 Division of Gastroenterology and Hepatology, Mayo Clinic, Rochester, MN 55905, USA

**Keywords:** obesity, gastrointestinal cancers, inflammation, insulin resistance, adipokines

## Abstract

Obesity is a global health crisis with profound implications for cancer risk, particularly within the gastrointestinal (GI) tract. Mounting evidence demonstrates that excess adiposity contributes to the initiation, progression, and poor outcomes of GI malignancies through a constellation of interrelated mechanisms. This review comprehensively examines the biologic pathways linking obesity to cancers of the esophagus, stomach, colon, liver, pancreas, and gallbladder. Chronic low-grade inflammation, driven by adipose tissue-derived cytokines and immune cell infiltration, plays a central role in tumorigenesis via the activation of NF-κB, STAT3, and other pro-oncogenic signaling cascades. Hyperinsulinemia and insulin resistance increase mitogenic IGF-1 signaling, while dysregulated adipokines, particularly elevated leptin and reduced adiponectin, promote cellular proliferation and impair tumor suppression. Dysbiosis of the gut microbiome and alterations in bile acid metabolism generate carcinogenic metabolites that contribute to DNA damage and immune evasion. Additionally, obesity-induced tissue hypoxia fosters tumor growth through HIF-1α-mediated pathways. We further highlight organ-specific associations, such as visceral adiposity’s role in Barrett’s esophagus and hepatocellular carcinoma emerging from metabolic dysfunction-associated steatotic liver disease (MASLD). Importantly, emerging data suggest that weight loss, achieved via lifestyle, pharmacologic, or surgical interventions, may mitigate these carcinogenic pathways and improve tumor biology. As obesity prevalence continues to rise globally, elucidating its mechanistic ties to GI malignancies is essential for risk stratification, prevention strategies, and personalized care. By integrating epidemiologic and molecular insights, this review underscores the need for multidisciplinary approaches to curb the oncogenic burden of obesity and improve outcomes in GI oncology.

## 1. Introduction

Obesity has emerged as a global epidemic with a rapidly increasing prevalence, affecting more than 40% of the United States population [1]. The rise is not only a reflection of changing dietary and physical activity patterns but also a marker of profound alterations in socioeconomic and environmental conditions worldwide [2]. Obesity is a well-established risk factor for a variety of chronic conditions, including cardiovascular disease, type 2 diabetes, and several forms of cancer [3]. In particular, the persistent excess of adipose tissue leads to systemic inflammation, insulin resistance, and dysregulated hormone secretion, contributing to the development and progression of malignancies [4,5,6].

Gastrointestinal cancers, encompassing colorectal, pancreatic, hepatic, esophageal, and other malignancies, represent a significant public health challenge, accounting for a substantial proportion of cancer incidence and cancer-related mortality [7]. Worldwide in 2022, an estimated 4.8 million individuals received a new diagnosis of gastrointestinal cancers, and approximately 3.2 million people died from these cancers [7]. Over recent decades, the incidence of these cancers has continued to rise, coinciding with the global surge in obesity rates [8]. A recently published meta-analysis of prospective cohort studies found that obesity significantly increases the risk of liver cancer (RR = 1.64, 95% CI: 1.47–1.84) and colorectal cancer (CRC; RR = 1.37, 95% CI: 1.24–1.50) [9].

Metabolic dysfunction associated with obesity, including chronic low-grade inflammation, oxidative stress, and altered adipokine levels, is increasingly recognized as a key driver in the pathogenesis of gastrointestinal cancers [10,11]. These metabolic alterations may create an environment that not only facilitates the initiation of carcinogenesis but also influences tumor progression and response to treatment [11].

Hence, understanding the link between obesity and gastrointestinal malignancies is critical for developing effective strategies to reduce cancer risk and improve patient outcomes. Weight loss, achieved through lifestyle modifications, pharmacotherapy, or procedural interventions (e.g., surgical or endoscopic), has been shown to positively impact metabolic parameters and reduce the risk of cancer [12,13,14]. Moreover, emerging evidence suggests that weight loss can lead to improvements in tumor biology, potentially altering the clinical course of gastrointestinal cancers [15]. By elucidating the association between obesity and these cancers, clinicians can better tailor preventive and therapeutic approaches to individual patients.

The objective of this review is to comprehensively explore the association between obesity and gastrointestinal cancers and to assess the impact of weight loss on cancer risk and outcomes. By synthesizing current epidemiological data and mechanistic insights, this review aims to clarify how interventions that reduce weight may alter cancer risk and progression, ultimately guiding future research and informing clinical practice to improve overall patient care.

## 2. Mechanisms Linking Obesity and Gastrointestinal Cancer

### 2.1. Chronic Inflammation and Cytokine Dysregulation

Obesity is associated with chronic low-grade inflammation, which can promote carcinogenesis as excess adipose tissue leads to the secretion of inflammation-related macrophages and adipokines such as interleukin (IL)-6, plasminogen activator inhibitor-1 (PAI-1), and C-reactive protein (CRP). Adipose tissue releases pro-inflammatory cytokines, enhancing tumor initiation, progression, and metastasis through the creation of a pro-inflammatory tumor microenvironment, immune evasion, and direct stimulation of cancer cell growth and survival. These molecules are secreted by macrophages with the M1 phenotype and play a large role in the transition between acute and chronic inflammation and inflammation-related gastrointestinal cancers [16,17]. Additionally, obesity-induced inflammation activates the NLRP3 inflammasome, leading to increased secretion of IL-1β and IL-18, further promoting pro-inflammatory cytokine dysregulation and a tumorigenic microenvironment [18]. The pro-inflammatory cytokines IL-1β and IL-36γ, often elevated in obesity, activate procarcinogenic signaling pathways such as the Wnt signaling cascade, leading to increased epithelial proliferation and tumorigenesis in the colon [19,20].

Similarly, tumor necrosis factor (TNF)-α and IL-6 are elevated in the colonic mucosa of individuals with obesity, activating procarcinogenic pathways like NF-κB and ERK 1/2, which promote cell proliferation and survival [19]. The NF-κB-IL-6-STAT3 signaling pathway promotes the proliferation and survival of tumor-initiating cells in the intestinal epithelium and protects them from apoptosis, thereby facilitating tumor growth and progression. TNF-α recruits immunosuppressive cells, which can inhibit anti-tumor immune responses and enhance tumor cell survival and metastasis [21]. For example, in colitis-associated cancers (CACs), IL-23 and its downstream cytokines IL-17 and IL-22 have also been shown to play critical roles in development and progression through chronic inflammation and support the survival and proliferation of malignant cells [22]. 

The chronic inflammatory state of individuals with obesity also leads to the overproduction of neutrophils, promoting carcinogenesis in the gastrointestinal tract through several mechanisms [23]. For instance, through the formation of neutrophil extracellular traps (NETs), neutrophils induce an inflammatory response in epithelial cells via Toll-like receptor 4 (TLR4)-dependent pathways, leading to the upregulation of pro-inflammatory cytokines such as IL-1β, IL-6, IL-8, and IL-1; this creates a tumor-promoting environment [24].

Additionally, in pancreatic ductal adenocarcinoma (PDAC), obesity-driven adipose tissue can act as a damage signal, leading to the accumulation of tumor-associated neutrophils (TANs) and active inflammasomes within the tumor microenvironment. Inflammasomes and TANs promote tumor progression through genomic instability and the activation of pro-tumorigenic pathways [25]. Furthermore, obesity can enhance the recruitment of immature myeloid cells and the TH17 immune response, accelerating carcinogenesis in the gastrointestinal tract. Ericksen and colleagues demonstrated the proliferation of immature myeloid cells and TH17 in models of Helicobacter-induced gastric cancer [26]. The interaction between neutrophils and the intestinal epithelium can also lead to the production of reactive oxygen species (ROS) and nitrates, which have mutagenic effects and can activate procarcinogenic signaling pathways such as NF-κB [27].

### 2.2. Insulin Resistance and Hyperinsulinemia

Insulin and insulin-like growth factor 1 (IGF-1) signaling play a role in carcinogenesis through multiple processes, which include the following: activation of PI3K/Akt/mTORC, and Raf/MAPK signaling pathways, upregulation of glucose transporters (e.g., GLUT1), initiation of key glycolytic enzymes (e.g., LDHA, LDH5, HK II, and PFKFB3), aberrant expression of the oncogenes (e.g., MYC and KRAS), and/or overexpression of signaling proteins (e.g., HIF-1, TGF-β1, PI3K, ERK, Akt, and mTOR) [27].

Obesity-induced hyperinsulinemia increases the bioavailability of IGF-1 by reducing the levels of its binding proteins (IGFBP-1 and IGFBP-2), thereby enhancing IGF-1’s mitogenic and anti-apoptotic effects [27]. The activation of IGF-1 signaling pathways has a significant role in obesity-related gastrointestinal cancer through its effects on cell proliferation, such as on intestinal epithelial cells. IGF-1 acts on the phosphatidylinositol 3-kinase (PI3K)/Akt and mitogen-activated protein kinase (MAPK)/p38 signaling pathways, which are necessary for cell survival, growth, and proliferation. These pathways can also lead to abnormal tissue renewal and function, contributing to carcinogenesis [28,29,30]. This imbalance in signaling pathways may lead to increased cell proliferation and reduced apoptosis, creating a favorable environment for tumor development. Furthermore, this cascade leads to the production of ROS via nicotinamide adenine dinucleotide phosphate (NADPH) oxidase isoforms, which can cause deoxyribonucleic acid (DNA) oxidation and genomic instability, promoting carcinogenesis [31]. Furthermore, insulin resistance in vascular endothelial cells can promote intestinal tumor formation. This is associated with the increased expression of vascular cell adhesion molecule-1 (VCAM-1) and leukocyte adhesion, creating a pro-inflammatory state that supports tumorigenesis [32].

### 2.3. Adipokine Imbalance

The dysregulation of adipokines, particularly leptin and adiponectin, is another mechanism of obesity leading to a risk of gastrointestinal cancer [33]. Leptin is secreted almost exclusively by white adipose tissue and circulates in blood serum in both free and bounded forms [34]. In individuals with obesity, the level of leptin in blood serum is higher than that in those with a lower body mass index (BMI), which can promote cancer cell proliferation, invasion, and metastasis through various signaling pathways similar to the ones discussed in previous sections. These pathways include JAK/STAT, MAPK, and PI3K/mTOR [34,35,36]. Paik et al. documented that leptin expression increased gradually in the normal–adenoma–adenocarcinoma sequence of CRC development and promoted their proliferation [37]. Additionally, Song and colleagues postulated that leptin could induce the expression of SIRT1 through the activation of the NF-E2-related factor 2 (Nrf2) pathway [38]. This upregulation of SIRT1 has been shown to stimulate the migration and invasion of colon cancer cells, thereby playing a role in colon carcinogenesis [38].

Leptin also activates the mTOR pathway in intestinal epithelial cells, leading to lipid droplet formation, cytokine production, and increased cell proliferation. This pathway is crucial for cellular growth and is frequently altered in tumors, indicating that leptin-induced mTOR activation may contribute to the enhanced susceptibility to CRC in individuals with obesity [39]. In gastric cancer, leptin has been shown to stimulate migration and invasion of cancer cells by activating the JAK-STAT and MEK pathways as discussed earlier. This activation also contributes to the maintenance of cancer and further metastasis [40].

In contrast to leptin, adiponectin generally has anti-tumorigenic effects, and its levels are often reduced in obesity, leading to an increased risk of gastrointestinal cancers through several mechanisms [41]. Nishihara and colleagues found that adiponectin-knockout mice demonstrated increased tumor size and progression, correlating with higher cell proliferation rates and elevated cyclooxygenase-2 (COX-2) expression in tumors [42]. Furthermore, adiponectin activates AMP-activated protein kinase (AMPK), which in turn inhibits the mTOR pathway, thereby reducing cell proliferation and tumor growth [42,43]. Moon et al. also demonstrated that, in adiponectin-deficient mice, there was increased inflammation and angiogenesis, critical in tumor development and progression [44]. The expression of adiponectin receptors (AdipoR1 and AdipoR2) is reduced in CRC tissues compared with that in normal tissues, impairing the anti-tumorigenic effects of adiponectin, contributing to cancer progression [45]. Adiponectin influences the expression of epithelial–mesenchymal transition (EMT) markers, which are involved in cancer metastasis. Lower adiponectin levels are associated with increased EMT, facilitating cancer cell invasion and metastasis [46].

### 2.4. Gut Microbiome Dysbiosis

Obesity can alter the gut microbiota, leading to dysbiosis, or an imbalance in the microbiota. Dysbiosis can result in the increased production of pro-inflammatory bacterial metabolites, secondary bile acids, DNA damage, and activation of oncogenic signaling pathways, damaging the intestinal epithelium and promoting carcinogenesis [47,48]. Research has found that, in the development of colon cancer, certain bacteria are more abundant in the intestinal microbiota: *Peptostreptococcus, Bacteroides, Porphyromonas, Streptococcus, Fusobacterium*, and *Parvimonas* [49,50,51]. Depending on the type of bacteria, different mechanisms are involved in promoting carcinogenesis. An example is *Clostridioides difficile,* which creates an enzymatic change of primary bile acids to secondary bile acids, mainly lithocholic acid and deoxycholic acid, which exhibit pro-inflammatory and carcinogenic activity [51]. Another well-studied example is a type of *Bacteroides fragilis* that can produce an enterotoxin, called enterotoxin-producing *B. fragilis* (ETBF). This bacterium causes the destruction of epithelium and γ-secretase-dependent E-cadherin cleavage, thereby increasing the permeability of the intestinal barrier and the signal transduction of E-cadherin and β-catenin in intestinal epithelial cells. Thus, proliferation and oncogenic transformation of CRC results [52]. Furthermore, *C. leptum* and *C. coccoides* can increase the amount of Treg cells, leading to the increased production of the anti-inflammatory cytokine IL-10 in mice; the upregulation of IL-10 causes immunosuppressive effects within the tumor microenvironment, facilitating tumor cell proliferation, invasion, and immune evasion [53].

In regard to gastric cancer, *Helicobacter pylori* infection is established to be a risk factor, as *Helicobacter pylori* induces chronic inflammation and promotes carcinogenesis [54]. Similarly, *Fusobacterium* species have been implicated in esophageal cancer [55], while *Porphyromonas gingivalis* and *Clostridium* species are associated with an increased risk of pancreatic cancer [56]. Newer studies are examining therapeutic interventions targeting the gut microbiome, such as probiotics, fecal microbiota transplantation (FMT), and microbiota-based treatments. The hope is that these interventions may target the microbiome and potentially prevent or treat gastrointestinal cancers [56].

In addition to bacteria increasing the risk of gastrointestinal cancers, the disruption of the intestinal epithelium, such as loss of its function and thinning of the mucus layer, can lead to a higher risk as it is the first barrier against the invasion of bacteria and other pathogens. A disrupted intestinal epithelium can lead to the transfusion of harmful bacterial products such as lipopolysaccharides (LPSs). Increased LPSs cause a systemic inflammatory response, in turn increasing the risk for the later development of CRC [57]. LPSs can also enhance tumor cell invasion and metastasis through various molecular pathways, including Toll-like receptor 4 (TLR4)/nuclear factor-κB (NF-κB) signaling [57,58]. This activation results in a chronic inflammatory environment that enhances and promotes tumorigenesis [59]. Moreover, LPS has been shown to increase tumor cell adhesion, invasion, and metastasis through the upregulation of the urokinase plasminogen activator (u-PA) system and the secretion of vascular endothelial growth factor-C (VEGF-C). These pathways promote lymphangiogenesis and lymphatic metastasis [60]. Additionally, LPS-induced inflammation creates NETs, which further exacerbate cancer progression and metastasis [61].

### 2.5. Bile Acid Metabolism Alterations

Obesity is associated with the dysregulation of bile acid metabolism, increasing levels of cytotoxic secondary bile acids (SBAs) [62]. SBAs are bile acids produced from primary bile acids through the action of gut bacteria in the colon; they are formed when bacteria deconjugate and dehydroxylate primary bile acids, such as cholic acid and chenodeoxycholic acid. Epidemiological data showed that the incidence of CRC is higher in patients with higher fecal bile acid concentrations. Research has found that this link between bile acid and CRC is through the following mechanisms: SBAs induce DNA damage, oxidative stress and inflammation and activates signaling pathways that promote cell proliferation and tumorigenicity, contributing to gastrointestinal cancer development [63,64]. According to a review article by Tsuei et al., gut dysbiosis and SBA dysregulation, frequently observed in patients with obesity, are also contributors to inflammation and injury of the liver and colon [48]. SBAs are hydrophobic and cytotoxic to colonic crypt epithelial cells. For example, the SBA deoxycholic acid (DCA) may be involved in the activation of protein kinase C (PKC) and the destruction of the cell membrane [65]. DCA has an anti-apoptotic effect in most cells and can induce the proliferation of colon epithelial cells and adenoma cells [62,64]. Further, DCA has been shown to induce ectodomain shedding of the EGFR ligand amphiregulin (AREG), which activates EGFR and downstream signaling pathways like MAPK and STAT3, promoting cell cycle progression and tumorigenicity in CRC and pancreatic ductal adenocarcinoma [66].

In addition, lithocholic acid (LCA), another SBA, can increase gastrointestinal cancer risk by stimulating the expression of micro-RNA-21 (miR-21) in CRC cells. The expression of miR-21 inhibits the tumor suppressor PTEN via the STAT3 and ERK-1/2 signaling pathways. Cell proliferation is then enhanced through the activation of the PI3K/AKT signaling pathway [67]. LCA also induces colonic epithelial cell cancer by promoting the muscarinic 3 receptor (M3R) and Wnt/β-catenin signaling pathways [68]. Cancer stem cells (CSCs) are then produced, playing a part in colon cancer development and progression. The ERK1/2 MAPK and STAT3 pathways promoted by LCA also stimulate IL-8 expression in CRC cells. Research has shown that the overexpression of IL-8 is associated with a poor prognosis in CRC and contributes to metastasis by promoting endothelial cell proliferation and tube-like formation [67]. In addition to all the aforementioned roles LCA has in gastrointestinal cancer, LCA also increases the expression of urokinase-type plasminogen activator receptor (uPAR), enhancing cell invasiveness in colon cancer cells through the MAPK and AP-1 signaling pathways [69].

### 2.6. Hypoxia

**Figure 1:**
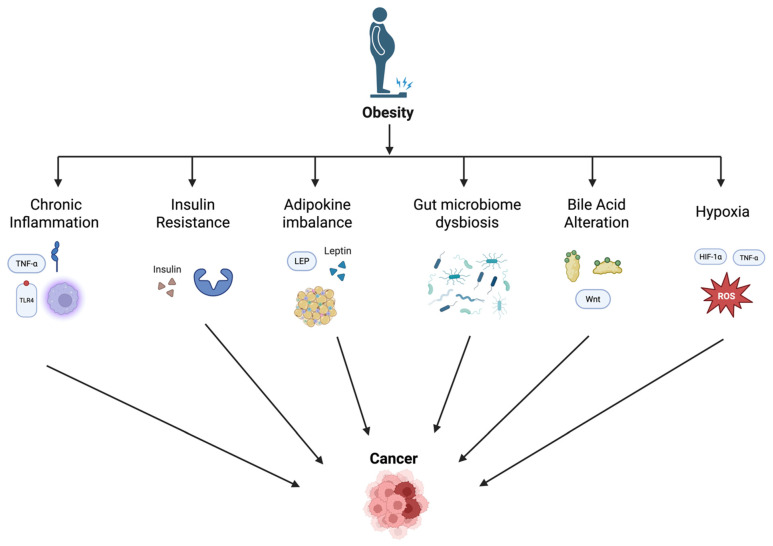
Mechanisms linking obesity to gastrointestinal cancers.

In individuals with obesity, excess adipose tissue often becomes hypoxic due to inadequate blood supply relative to increased tissue mass. Local hypoxia, in turn, creates hypoxia-inducible factors (HIFs), particularly HIF-1α; this promotes a pro-inflammatory and pro-tumorigenic environment through angiogenesis, metabolic reprogramming, and other processes that support tumor growth and survival [70]. Hypoxia also leads to the generation of ROS, which create oxidative stress and DNA damage, well-known critical steps in the initiation of cancer. Hypoxia in adipose tissue induces the release of pro-inflammatory cytokines and adipokines, such as TNF-α and IL-6, contributing to chronic low-grade inflammation. This inflammatory state can promote carcinogenesis by inducing DNA damage, enhancing cell proliferation, and inhibiting apoptosis [5]. For example, in esophageal adenocarcinoma, obesity-related hypoxia exacerbates gastroesophageal reflux disease (GERD), a known risk factor for this type of cancer. Chronic inflammation, oxidative stress, and DNA damage in the esophageal epithelium due to hypoxia further contribute to cancer progression [71]. Moreover, HIF-1α activation can alter metabolic pathways, promoting glycolysis and angiogenesis, which support tumor growth and survival in the hypoxic tumor microenvironment, leading to the progression of gastrointestinal cancers in individuals with obesity [70]. In CRC, for example, hypoxia-induced inflammation and altered insulin signaling pathways lead to increased insulin resistance and hyperinsulinemia, which can promote colorectal carcinogenesis through the insulin/IGF axis as stated previously [72]. A summary of the main mechanisms linking obesity to GI cancers is presented in Figure 1.

### 2.7. Other Mechanisms

In addition to these well-established mechanisms, emerging evidence highlights the role of epigenetic modifications, including DNA methylation and histone acetylation, in mediating the effects of obesity on gastrointestinal tumorigenesis. These changes may influence gene expression in pathways related to inflammation, metabolism, and cell cycle regulation [73]. Moreover, sex-specific differences, such as hormonal influences in gallbladder cancer, variations in visceral fat distribution, and obesity-associated shifts in sex hormone levels (e.g., increased estrogen, decreased sex hormone-binding protein) may also contribute to differential cancer susceptibility and progression and warrant further investigation [74].

Beyond hormonal and epigenetic mechanisms, genetic evidence has increasingly supported a causal role of obesity in GI cancers. Mendelian randomization (MR) studies have demonstrated that genetically predicted BMI is associated with an increased risk of colorectal and esophageal adenocarcinoma and pancreatic cancers. These studies minimize confounding and reverse causation and suggest that the carcinogenic effect of obesity may be even greater when considering lifelong adiposity, as opposed to BMI measured at a single time point [75].

Furthermore, genome-wide association studies (GWASs) have identified multiple loci linking obesity and cancer risk. Notably, polymorphisms in the *FTO* gene (e.g., rs8050136 and rs9939609) have been associated with both elevated BMI and increased colorectal cancer risk. A large Japanese cohort study reported a significant interaction between *FTO* risk alleles and circulating adiponectin levels, highlighting a synergistic role for genetic and hormonal pathways in obesity-related colorectal tumorigenesis [76].

## 3. Obesity and Specific Gastrointestinal Cancers

### 3.1. Esophageal Adenocarcinoma

GERD, obesity, and Barrett’s esophagus (BE) are well-established, interrelated risk factors for the development of esophageal adenocarcinoma (EAC) [77]. GERD can chronically inflame the stratified squamous mucosa of the distal esophagus, which can then heal abnormally into an intestinal-type columnar mucosa known as BE. Consequently, BE is a major risk factor for EAC [77]. For a long time, obesity was thought to worsen GERD via abdominal fat mechanically increasing intra-abdominal pressure. This pressure was thought to lead to a rise in the gastroesophageal pressure gradient that then exaggerated acid reflux. However, recent data has shown that visceral adipose tissue (VAT) also plays a reflux-independent role in contributing to esophageal inflammation via the production of pro-inflammatory cytokines that can disrupt the gastroesophageal mucosa [77]. Increased leptin and decreased adiponectin, which are adipokines secreted by visceral fat, are independent risk factors for EAC progression [78].

One meta-analysis showed that, for each 5 kg/m^2^ increase in BMI, there was a strong association with EAC in both men and women [79]. Another study found that there is a strong association between BMI ≥ 40 kg/m^2^ and EAC compared with that at BMI < 25 kg/m^2^. This association was true in patients with and without GERD symptoms [80]. Another meta-analysis found that obesity is significantly associated with an increased risk for GERD symptoms, erosive esophagitis, and EAC in a dose-dependent fashion [81].

### 3.2. Gastric Cancer

Gastric cancer can be divided into cardia and non-cardia cancer, where 80–90% of non-cardia gastric cancer cases are associated with *H. pylori* infection [82]. *H. pylori* directly affect gastric epithelial cells through protein modification and gene mutation and indirectly by promoting inflammatory changes to the gastric mucosa [82]. In contrast, obesity has been identified as a significant risk factor for cardia gastric cancer [19]. The mechanism for this relationship is likely multifactorial [19]. One proposed pathway is that obesity promotes acid reflux, which increases the risk for BE, and subsequently increases the risk of cardia gastric cancer. To further elaborate, obesity contributes to a higher risk of GERD by elevating intra-abdominal pressure, which can cause displacement and dysfunction of the lower esophageal sphincter; by raising the likelihood of hiatal hernia; and by increasing the secretion of bile and pancreatic enzymes [19]. Obesity is also associated with decreased adiponectin, increased leptin, increased insulin resistance, and a pro-inflammatory state, which synergistically promotes carcinogenesis [83].

One meta-analysis found that increased BMI was positively associated with the risk of gastric cardia cancer with a summary relative risk of 1.21 for overweight and 1.82 for obesity. On the other hand, BMI was not found to be associated with non-cardia gastric cancer [84]. Another meta-analysis demonstrated a positive, though weak, association between increased BMI and cardia cancer in studies looking at the U.S. and European population but no clear association in studies from China [85]. Yet, another study in a Chinese province found increasing cardia gastric cancer and declining non-cardia gastric cancer, suggesting environmental factors like alcohol consumption, low vegetable intake, and socioeconomic differences may contribute [23]. This is a trend that has also been observed globally, underscoring the need to distinguish between the two types of gastric cancer and further investigating their unique risk factors [86].

### 3.3. Colorectal Cancer (CRC)

The literature suggests that there is a strong, positive association between CRC and obesity. The association has generally been found to be stronger for colon than for rectal cancer [87]. Several mechanisms have been proposed for this association. First, like for many of the cancers described above, obesity has a significant effect on the insulin level, pro-inflammatory cytokines, and adipokines, which can affect CRC risk [88]. Furthermore, an unbalanced diet consisting of highly processed food, high meat consumption, and low fiber intake can disrupt the microbiota, resulting in the production of harmful metabolites like ammonia, amines, and hydrogen sulfide that can induce inflammation and promote carcinogenesis [89]. In addition, metabolic syndrome has been associated with about a 13% increased risk of CRC [90]. In one prospective study in Korea, meeting three or more of the five criteria of metabolic syndrome, specifically the co-existence of abdominal obesity, glucose intolerance, and decreased HDL, showed the strongest association with the development of CRC [91]. Another meta-analysis showed that, as the number of metabolic syndrome components increased, the risk of CRC also seemed to increase, with waist circumference and hyperglycemia potentially having the largest impact on this association [92]. This association may be due to visceral adipose tissue being closely related to insulin resistance, thereby resulting in a state of chronic inflammation and promoting a carcinogenic environment that is responsible for the development of CRC [88].

### 3.4. Hepatocellular Carcinoma (HCC)

Metabolic dysfunction-associated steatotic liver disease (MASLD), previously known as NAFLD (i.e., nonalcoholic fatty liver disease), includes patients who have hepatic steatosis and have at least one of five cardiometabolic risk factors. There is a strong association between obesity and increased risk of HCC, either independently or as a cofactor with other liver disease [93]. In obesity, hepatocytes store extra lipids, mainly as triglycerides, which leads to simple steatosis (SS), the initial stage of MASLD. If SS is not managed properly, the liver is infiltrated by immune cells that produce cytokines and interleukins that can lead to a chronic intrahepatic inflammatory process that can then progress to metabolic dysfunction-associated steatohepatitis (MASH). Prolonged inflammation results in fibrogenesis or the formation of scar tissue, where the liver is significantly damaged and can then progress to cirrhosis, which is a significant risk factor for HCC [94].

One study found that, in a group of 90,000 American adults, the risk of dying from HCC was 4.5 times higher in men with a BMI of 35 kg/m^2^ or above than in men with a healthy BMI of <25 kg/m^2^ [95]. Another study found that weight, BMI, waist and hip circumference, and weight change per year were all associated with a higher risk of HCC. Weight gain can increase the risk of HCC by up to 2.5-fold [96]. Obesity can also have a synergistic effect on HCC in patients with viral infections. In one Taiwanese study, obesity (i.e., BMI ≥ 30 kg/m^2^) was independently associated with a four-fold risk of HCC among anti-hepatitis C virus (HCV) seropositive patients, as well as a two-fold risk in persons without a history of hepatitis B virus (HBV) or HCV infections when controlling for metabolic factors. Importantly, there was a 100-fold increased risk among HBV and HCV carriers with both obesity and diabetes [97].

### 3.5. Pancreatic Cancer

The well-established, modifiable risk factors for the development of pancreatic cancer are obesity, type 2 diabetes, and smoking [33]. In obesity, adipose tissues release free fatty acids, which can enter the circulation and accumulate in organs like the pancreas [98]. Fatty infiltration of the pancreas has been associated with the development of pancreatic intraepithelial neoplasia, which are precursors to the development of pancreatic ductal adenocarcinoma [99]. In addition, studies suggest that a high-fat diet and resistant starch can alter the gut microbiome, promoting the release of inflammatory cytokines and triggering cancer cell proliferation through the MAPK signaling pathway, potentially leading to pancreatic cancer progression [98]. Furthermore, the disruption of metabolic and inflammatory states can lead to reduced insulin tolerance and hyperinsulinemia, which then directly stimulate cell growth by binding on target cells like the pancreatic tissue. In addition, insulin can increase the production of IGF-1, which has been shown to promote pancreatic cell proliferation [100].

One meta-analysis found there was a small positive increase in the risk of pancreatic cancer per unit increase in BMI and that individuals with obesity have a 19% higher risk than those with a normal BMI [101]. Another meta-analysis demonstrates that the relative risk of pancreatic cancer per 5 kg/m^2^ increase in BMI was 1.12 in both males and females [102]. One pooled analysis found that a higher BMI increased the risk of pancreatic cancer, and this relationship appears to be independent of age, sex, smoking, physical activity, and diabetes [103]. Some studies, however, have found a significant association with central adiposity but not with BMI [38]. Other studies have found an increased waist circumference to be positively associated with pancreatic cancer in women but not in men [103].

### 3.6. Gallbladder Cancer

Studies show a statistically significant relationship between obesity and increased risk of gallbladder cancer [39]. Obesity is a well-known risk factor for gallstone formation [39]. In fact, gallstones are a major risk factor for gallbladder cancer, possibly due to the chronic trauma they inflict on the gallbladder mucosa, which can lead to dysplasia [104]. Hyperinsulinemia, which is closely linked to obesity, has been shown to increase hepatic cholesterol secretion and cholesterol supersaturation leading to gallstone formation [39]. Some studies have also suggested that, in patients with obesity, the gallbladder is less sensitive to cholecystokinin, delaying postprandial gallbladder emptying [104]. Furthermore, obesity can affect the sex hormone balance, particularly causing an excess presence of unregulated estrogen [104]. Estrogen can then cause an increase in cholesterol secretion, as well as a reduction in bile salt secretion [105].

One meta-analysis found that the summary relative risk of gallbladder cancer was significantly higher for both overweight individuals and for individuals with obesity compared to those with a BMI < 25 kg/m^2^, and this association was significantly stronger for women compared to men [106]. Another meta-analysis found similar results, again finding a higher summary relative risk of gallbladder cancer for overweight individuals and individuals with obesity than for patients with a BMI between 18 and 25 kg/m^2^ [105]. The mechanisms linking obesity to specific GI cancers are presented in Table 1.

### 3.7. Limitations

This review is narrative in nature and thus subject to inherent limitations. This review is intended as a comprehensive narrative synthesis and is therefore not structured according to systematic review methodology. As such, it may be subject to selection bias in the studies included. While we cover a broad range of mechanistic pathways linking obesity to gastrointestinal cancers, such as inflammation, insulin resistance, adipokine imbalance, microbiome alterations, bile acid dysregulation, and hypoxia, the strength and depth of evidence supporting each mechanism vary by cancer type. Some associations, particularly those involving gastric and gallbladder cancers, are less well established compared to colorectal or hepatic malignancies. Additionally, emerging areas such as epigenetics, microbiota-derived metabolites, and genetic predisposition are highlighted, but data in these domains remain incomplete and evolving. Finally, the multifactorial nature of both obesity and gastrointestinal cancer risk makes it difficult to isolate the contribution of individual pathways with certainty.

## 4. Conclusions

In conclusion, obesity is a multifaceted risk factor that contributes to the development and progression of gastrointestinal cancers through a complex interplay of inflammatory, metabolic, hormonal, and microbial mechanisms. This review underscores the biologic plausibility and epidemiologic evidence linking excess adiposity to malignancies such as colorectal, gastric, esophageal, hepatic, pancreatic, and gallbladder cancers. As the prevalence of obesity continues to rise globally, addressing its oncogenic effects is an urgent public health priority. Understanding the underlying mechanisms offers a critical opportunity to inform cancer prevention strategies, guide risk stratification, and develop targeted therapeutic interventions. Future research should focus on clarifying causal pathways, identifying high-risk phenotypes, and evaluating the impact of metabolic modulation on cancer incidence and outcomes.

## Figures and Tables

**Table 1:** Mechanisms linking obesity to specific gastrointestinal cancers.

Gastrointestinal Cancer	Mechanisms Linking Obesity to Cancer
Esophageal Adenocarcinoma	GERD and Barrett’s esophagus progression; VAT-induced cytokine inflammation; altered adipokines (↑ leptin, ↓ adiponectin)
Gastric Cancer (Cardia)	GERD-induced mucosal damage; elevated intra-abdominal pressure; insulin resistance; ↑ leptin and inflammation
Colorectal Cancer	Insulin resistance, adipokine imbalance, chronic inflammation; dietary effects on microbiota; metabolic syndrome
Hepatocellular Carcinoma	Lipid accumulation → MASLD/MASH → fibrosis and cirrhosis; inflammatory cytokine infiltration
Pancreatic Cancer	Free fatty acid infiltration; insulin resistance and hyperinsulinemia; gut microbiome-mediated inflammation
Gallbladder Cancer	Gallstone formation from cholesterol supersaturation; delayed gallbladder emptying; hormonal changes (↑ estrogen)
